# Insights into *in vivo* adipocyte differentiation through cell-specific labeling in zebrafish

**DOI:** 10.1242/bio.058734

**Published:** 2021-09-09

**Authors:** Paola Lepanto, Florencia Levin-Ferreyra, Uriel Koziol, Leonel Malacrida, José L. Badano

**Affiliations:** 1 Human Molecular Genetics Lab, Institut Pasteur de Montevideo, Montevideo, Mataojo 2020, CP11400, Uruguay; 2 Sección Biología Celular, Facultad de Ciencias, Universidad de la República, Montevideo, Igua 4225, CP11400, Uruguay; 3 Advanced Bioimaging Unit, Institut Pasteur de Montevideo and Universidad de la República, Montevideo, Mataojo 2020, CP11400, Uruguay; 4 Departamento de Fisiopatología, Hospital de Clínicas, Facultad de Medicina, Universidad de la República, Montevideo, Av. Italia s/n, CP11600, Uruguay

**Keywords:** Zebrafish, Adipocyte, Nile Red, Blood vessels

## Abstract

White adipose tissue hyperplasia has been shown to be crucial for handling excess energy in healthy ways. Though adipogenesis mechanisms have been underscored *in vitro*, we lack information on how tissue and systemic factors influence the differentiation of new adipocytes. While this could be studied in zebrafish, adipocyte identification currently relies on neutral lipid labeling, thus precluding access to cells in early stages of differentiation. Here we report the generation and analysis of a zebrafish line with the transgene *fabp4a(-2.7):EGFPcaax*. *In vivo* confocal microscopy of the pancreatic and abdominal visceral depots of transgenic larvae, revealed the presence of labeled mature adipocytes as well as immature cells in earlier stages of differentiation. Through co-labeling for blood vessels, we observed a close interaction of differentiating adipocytes with endothelial cells through cell protrusions. Finally, we implemented hyperspectral imaging and spectral phasor analysis in Nile Red-labeled transgenic larvae and revealed the lipid metabolic transition towards neutral lipid accumulation of differentiating adipocytes. Altogether our work presents the characterization of a novel adipocyte-specific label in zebrafish and uncovers previously unknown aspects of *in vivo* adipogenesis.

This article has an associated First Person interview with the first author of the paper.

## Abstract (precis)

**Summary:** Analysis of the differentiation of adipocytes *in vivo* through cell-specific labeling in zebrafish, revealed their early interaction with blood vessels as well as early lipid metabolic changes.

## INTRODUCTION

White adipose tissue (WAT) is present in mammals as well as in the other vertebrates, in the form of anatomically and functionally distinct depots (Zwick et al., 2018). It is formed by adipocytes, adipocyte precursors and macrophages surrounded by a collagen-rich extracellular matrix, and is highly vascularized and innervated. In humans, both visceral and subcutaneous central WAT depots primarily play energy storage and endocrine functions, which are of central importance in the regulation of energy homeostasis. Thus, WAT dysfunction, which is usually associated with obesity, contributes to the development of metabolic syndrome associated-diseases such as type II diabetes, dyslipidemia and non-alcoholic fatty liver disease (Longo et al., 2019). Meanwhile, other depots such as those in the dermis, bone marrow and mammary gland, contribute to regulation of local innate immunity and to the repair of adjacent tissues (Zwick et al., 2018).

In humans, the localization and mode of remodeling of adipose tissue have been associated with healthy or pathological phenotypes (Hepler and Gupta, 2017). Macroscopically, the expansion of subcutaneous WAT (SAT) is considered healthier than the growth of visceral WAT (VAT), as well as its localization in peripheral (extremities) versus central (abdomen in men; abdomen and hips in women) depots. Also, the accumulation of fat can occur in previously existing mature adipocytes (hypertrophy) or in newly differentiated cells (hyperplasia). Importantly, different lines of evidence support an association between adipose tissue hyperplasia with a healthier state as compared to hypertrophy (Vishvanath and Gupta, 2019). Adipogenesis is the process whereby stem cell-like precursors become committed into pre-adipocytes, which then differentiate into mature adipocytes. While initial formation of adipogenic progenitors occur in hematopoietic tissues (Hudak et al., 2014), adult progenitors reside locally associated to blood vessels of adipose tissue (Hilgendorf et al., 2019; Tang et al., 2008). Tissue environment and cellular composition may influence the differentiation of these locally residing progenitors (for example see Schwalie et al., 2018). Thus, the study of adipogenesis and its relationship with other elements in the tissue *in vivo*, is critical to understand normal and pathological processes.

Work on cultured cells has provided key information about transcriptional regulation of adipogenesis (Bahmad et al., 2020). Meanwhile studies in mice have been conducted to analyze the developmental origin of adipocyte progenitors (Hepler and Gupta, 2017). More recently, the use of zebrafish to analyze adipose tissue biology has captured attention as it promises to enable the study of the tissue and its cellular biology *in vivo*. Zebrafish develops only white adipose tissue, which first appears in visceral depots in early larval stages (Flynn et al., 2009; Minchin and Rawls, 2017b). In contrast, mice develop first SAT depots in embryonic stages while VAT appears postnatally (Hudak et al., 2014). Importantly, however, zebrafish adipocytes show the same subcellular characteristics, gene expression patterns and final distribution (visceral and subcutaneous) as in mammals (Flynn et al., 2009). Moreover, it has been reported that factors affecting body fat distributions in humans have comparable effects in zebrafish (Loh et al., 2020; Minchin et al., 2015). Thus, taking advantage of its fast external development and optical transparency, zebrafish is an ideal system to study cellular and tissular aspects of WAT development.

Current methods to label adipose tissue *in vivo* rely on the use of lipophilic dyes such as LipidTOX or Nile Red (Minchin and Rawls, 2017a). Nile Red is particularly useful because its absorption and emission spectral characteristics are modified according to the polarity of the environment surrounding the probe (Greenspan and Fowler, 1985). The emission of Nile Red in the context of neutral lipids is blue-shifted in comparison to when it is in the presence of polar lipids. This spectroscopic characteristic has been extensively used to label lipid droplets and to estimate the amount of adipose tissue in live larvae as well as to classify depots (Minchin and Rawls, 2017b). However, Nile Red stains all cell membranes, including the endoplasmic reticulum in which biogenesis of lipid droplets takes place (Olzmann and Carvalho, 2019). Lipid stores are composed of neutral lipids such as triacylglycerols and sterol esters, while polar lipids are present during droplet formation as well as during lipolysis. Maulucci et al. developed an approach to generate a lipid metabolic index using hyperspectral imaging of Nile Red and spectral phasor analysis (Di Giacinto et al., 2018; Maulucci et al., 2018). Thus, this method allowed them to differentiate among cells forced to carry out lipid movement (lipid storage or lipolysis) and those in a resting state. The application of this method to live larvae as well as the study of the interaction of adipocytes with other cells in the tissue would require to specifically label adipocytes independently of their fat load.

To address this issue we decided to generate an adipocyte specific reporter zebrafish line. Up to date, no factor has been identified that is expressed in all fat depots in mice (Cleal et al., 2017). However, there are several genes that are upregulated in adipocytes in different stages during differentiation, like those used extensively in cell culture of mammalian cells to monitor differentiation progress (Tang and Lane, 2012). As expected, much less information is available from zebrafish. We therefore selected early and late genes that are commonly used as adipocyte differentiation markers in mammalian cell culture models and with previous evidence of being expressed in zebrafish adipose tissue (Imrie and Sadler, 2010), cloned their putative promoter regions and generated transgenesis constructs. We show here that a *fabp4a* (previously called *fabp11a*) −2.7 kb proximal genomic region from zebrafish effectively drives the expression of EGFP in adipocytes both previous to and during the accumulation of fat. Membrane tagging of EGFP allowed us to observe the early interaction of adipocytes with blood vessels through adipocyte membrane protrusions. Furthermore, we adapted the method of Maulucci et al. by incorporating a three-component analysis in the phasor plot, which enabled us to analyze the lipid metabolism of EGFP-positive cells in live larvae before the formation of lipid droplets. Thus, this new zebrafish transgenic line is a valuable tool which will open new possibilities to study adipocytes and adipose tissue biology *in vivo*.

## RESULTS

### *fabp4a(-2.7):EGFPcaax* transgene is expressed in early and mature adipocytes

**Fig. 1.**
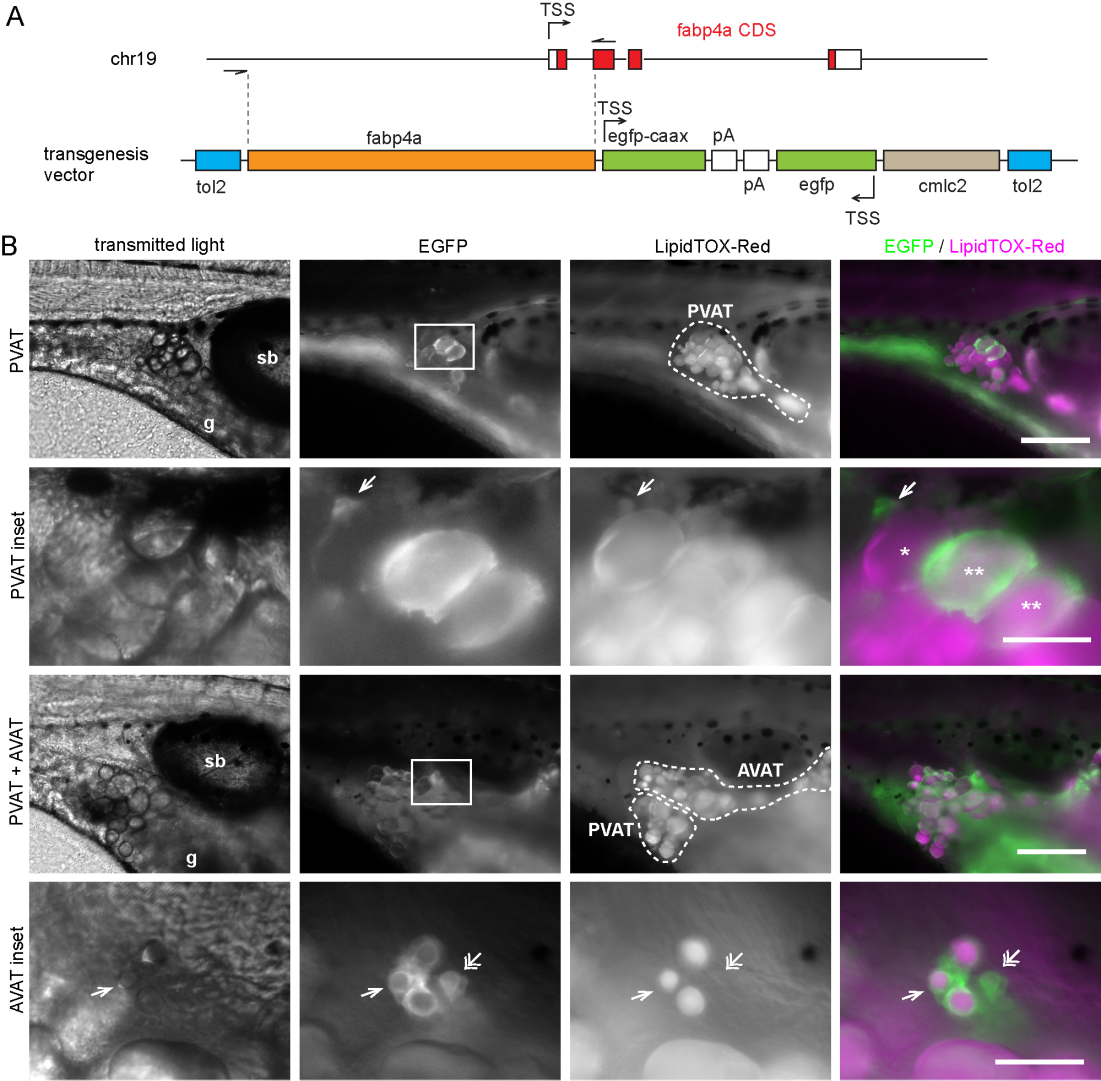
***fabp4a(-2.7):EGFPcaax* is expressed in early and mature adipocytes.** (A) The upper scheme shows the endogenous *fabp4a* gene in chromosome 19 with the transcription start site (TSS), exons (boxes), introns, and the coding sequence (CDS) in red. The cloned region is denoted between dashed lines. The lower scheme represents the vector used for transgenesis (tol2: tol2 sites; pA: SV40_late_polyA; cmlc2: cardiac myosin light chain 2 upstream region). (B) Epifluorescence microscopy images of live *fabp4a(-2.7):EGFPcaax* larvae from the incross of the F3 generation labeled with LipidTOX-Red. EGFP+ cells were present in the PVAT and AVAT depots. Asterisks indicate mature adipocytes stained with LipidTOX-Red with low (*) or high EGFPcaax expression (**). Single arrows denote early adipocytes expressing EGFPcaax with small lipid droplets. Double arrows indicate early adipocytes with EGFPcaax expression without LipidTOX-Red staining. sb, swim bladder; g, gut. Scale bars: B: panoramic views: 200 µm; insets: 50 µm.

Based on previously reported data on cell culture models and expression patterns in zebrafish we selected four different genes to work with: *adipoqb*, *cebpα*, *cfd*, *fabp4a*. All of them were previously reported to be expressed in zebrafish adipose tissue in larvae and/or adults (Imrie and Sadler, 2010). Taking into account our analysis of the promoter regions, we cloned approximately 2 kb of the proximal part of the zebrafish promoter for each gene (see Materials and Methods), and generated transgenesis constructs using the Tol2 system bearing the cardiac light chain myosin reporter gene, *cmlc2:GFP*, as an early selection marker (Fig. 1A). These constructs were injected together with mRNA coding for Tol2 transposase in the cytoplasm of one-cell stage embryos. Twenty-four hours post-fertilization (hpf) embryos with GFP expressing-cells in their hearts were selected for further breeding. We then analyzed larvae of 15–21 days post-fertilization (dpf) in the stereomicroscope, and observed the presence of labeled cells for the constructions with *cebpα* and *fabp4a* promoters. However, only in the latter case the EGFPcaax signal coincided with lipid droplets in mature adipocytes recognizable through transmitted light. Moreover, only in the case of larvae injected with the *fabp4a(-2.7):EGFPcaax* construct we observed mature adipocytes labeled along several generations (Fig. 1B). Therefore, we decided to continue working only with the *fabp4a(-2.7):EGFPcaax* line.

First, to assess EGFP expression in live *fabp4a(-2.7):EGFPcaax* larvae we stained individuals of different stages with the lipophilic dye LipidTOX-Red and analyzed them using epifluorescence microscopy. We observed EGFPcaax signal in the surface of mature adipocytes, both in the pancreatic and abdominal depots (PVAT and AVAT, respectively) (Fig. 1B, asterisks). Of note, expression levels varied among cells and this effect remained even after several outcrosses with the wild-type fish line. We also analyzed other depots present in 27 dpf larvae (renal VAT, ocular SAT, lateral SAT and anal fin ray SAT) and detected cells with expression of EGFPcaax (Fig. S1). Interestingly, besides mature adipocytes with readily visible lipid droplets, we observed EGFPcaax-positive (EGFP+) cells that had smaller lipid accumulations as well as cells that had no detectable LipidTOX-Red signal (Fig. 1B, single and double arrows, respectively). Based on the fact that mammalian *fabp4* is expressed during adipocyte differentiation (Tang and Lane, 2012), these results indicated that EGFP+ cells with no visible lipid droplets (to which we refer as EGFP+/LD− from now on) were likely adipocytes at initial stages of differentiation.

### *fabp4a(-2.7):EGFPcaax* transgene expression pattern recapitulates the adipose tissue expression domain of endogenous *fabp4a*

Embryonic *fabp4a* expression has been reported to be restricted to the lens, midbrain and the blood vessels of the head and trunk in 48 hpf embryos (Liu et al., 2007). Meanwhile, 15 dpf larvae present *fabp4a* expression in trunk vessels and in early adipocytes (Flynn et al., 2009). To determine the expression pattern of the *fabp4a(-2.7):EGFPcaax* transgene, we analyzed several developmental stages and compared it with endogenous *fabp4a* expression. For this, endogenous *fabp4a* expression was assessed using fluorescent wholemount *in situ* hybridization (WMISH), and *fabp4a(-2.7):EGFPcaax* transgene expression through immunolabeling with an anti-GFP antibody.

First, we checked the specificities of WMISH probes using *fli1:EGFP* transgenic embryos, which express EGFP in blood vessels, useful as an anatomical reference. For that, 2 dpf wild-type embryos were fixed and processed for WMISH as described in the Materials and Methods section. As specificity controls, we used the *fabp4a* sense probe (negative control) and an antisense probe for *slit2* (additional specificity control). To detect EGFP, we performed a final immunolabeling step. We observed a clear signal corresponding to *fabp4a* transcripts when the WMISH was performed with the antisense probe, which co-localized with *fli1:EGFP* immunodetection almost completely (Fig. S2; also note the presence of brain cells positive for *fabp4a* antisense probe without *fli1:EGFP* labeling). Neither of the other two probes generated similar patterns: no specific signal was observed with the *fabp4a* sense probe while the *slit2* antisense probe labeled the ventro-medial part of the neural tube as reported previously (Davison and Zolessi, 2021). These results corroborated the specificity of the *fabp4a* antisense probe and validated the post-WMISH immunofluorescence procedure and reagents.

**Fig. 2.**
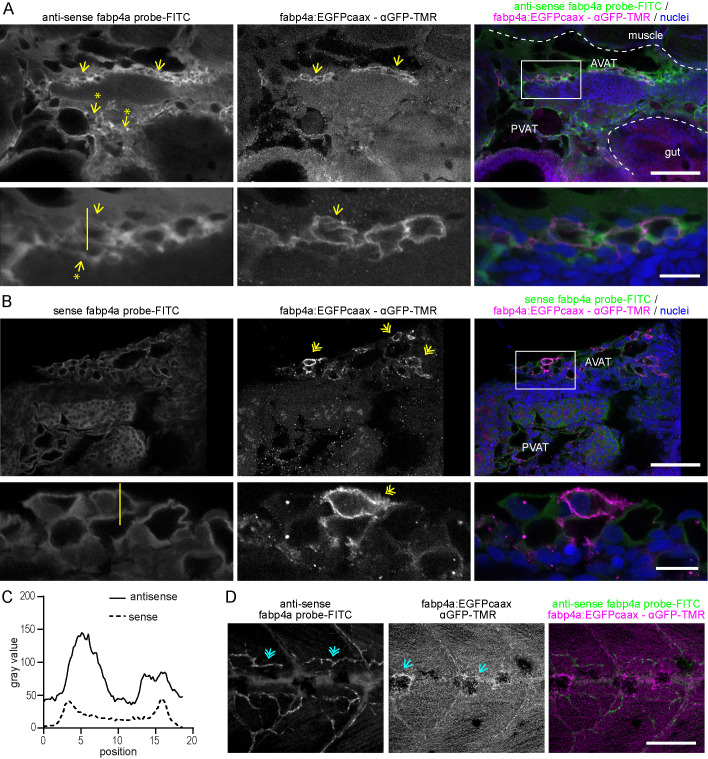
**Comparison of the expression pattern of *fabp4a(-2.7):EGFPcaax* and endogenous *fabp4a* mRNA in larva.** Images of *fabp4a(-2.7):EGFPcaax* larvae of 21 dpf processed for WMISH, immunolabeled with anti-GFP and analyzed *in toto* through confocal microscopy. (A) Panoramic (upper row) and magnified image (lower row) of the abdominal region of a larva labeled with *fabp4a* antisense probe. Yellow arrows denote the coincidence of EGFPcaax (immunofluorescence) and WMISH signal. Arrows with asterisks show regions with WMISH labeling and no EGFP signal. (B) Panoramic (upper row) and magnified image (lower row) of the abdominal region of a larva labeled with *fabp4a* sense probe. Yellow double arrows indicate regions with EGFPcaax signal without WMISH labeling. (C) Intensity profile of FITC fluorescence along the lines indicated in A and B insets. (D) Images of the trunk of a larva labeled with *fabp4a* antisense probe. Blue single arrows indicate pigment cells with EGFPcaax signal. Blue double arrows show WMISH labeling in blood vessels. Scale bars: A and B: 100 μm (upper row), 20 μm (lower row); D: 100 μm.

To compare the distribution of transgenic *fabp4a(-2.7):EGFPcaax* expression with that of endogenous *fabp4a* in larvae, we performed WMISH in individuals of 21 dpf, immunolabeled them with anti-GFP and analyzed them *in toto* using confocal microscopy. Endogenous expression was observed in the PVAT and AVAT areas with the *fabp4a* antisense probe, localizing within cells with EGFPcaax expression (Fig. 2A). While signal was observed with the *fabp4a* sense probe, likely due to the thickness of the sample hindering an efficient clearance of non-specific labeling during washing steps, it showed less fluorescence intensity than the anti-sense probe and a different subcellular localization (Fig. 2B,C). Also, EGFPcaax expression was evidenced in pigment cells in live embryos (Fig. S3). This expression pattern was not described before for endogenous *fabp4a* and, in larvae processed for WMISH, expression of endogenous *fabp4a* was not observed in superficial pigment cells (Fig. 2D, single arrows). Instead, we did observe staining for endogenous *fabp4a* in blood vessels (Fig. 2D, double arrows). In conclusion, the expression pattern of the transgene recapitulates the endogenous pattern of *fabp4a* in the adipose tissue, but not within blood vessels or in the brain.

### Development of early adipocytes *in vivo* and their relationship with blood vessels

Our results therefore supported the hypothesis that the *fabp4a(-2.7):EGFPcaax* transgene marks adipocytes during differentiation. To test this possibility we analyzed live larvae and embryos of different stages using confocal microscopy. First, we analyzed embryos in search of early expression of *fabp4a(-2.7):EGFPcaax*. *In vivo* confocal analysis of 2 dpf and 5 dpf embryos showed expression of EGFPcaax in cells along the antero-posterior axis at dorsal, lateral and ventral positions, all reminiscent of pigment cells (Fig. S3A,C, double arrows). The presence of early labeling of pigment cells suggested that this expression domain corresponded to ectopic expression of *fabp4a(-2.7):EGFPcaax* in the transgenic embryos. To discard autofluorescence or dispersion of light by pigments, we immunostained fixed embryos with anti-GFP antibody. We observed immunostaining co-localizing with EGFPcaax fluorescence (Fig. S3B,D, double arrows), thus confirming that in our fish line pigment cells are labeled by *fabp4a(-2.7):EGFPcaax*.

**Fig. 3.**
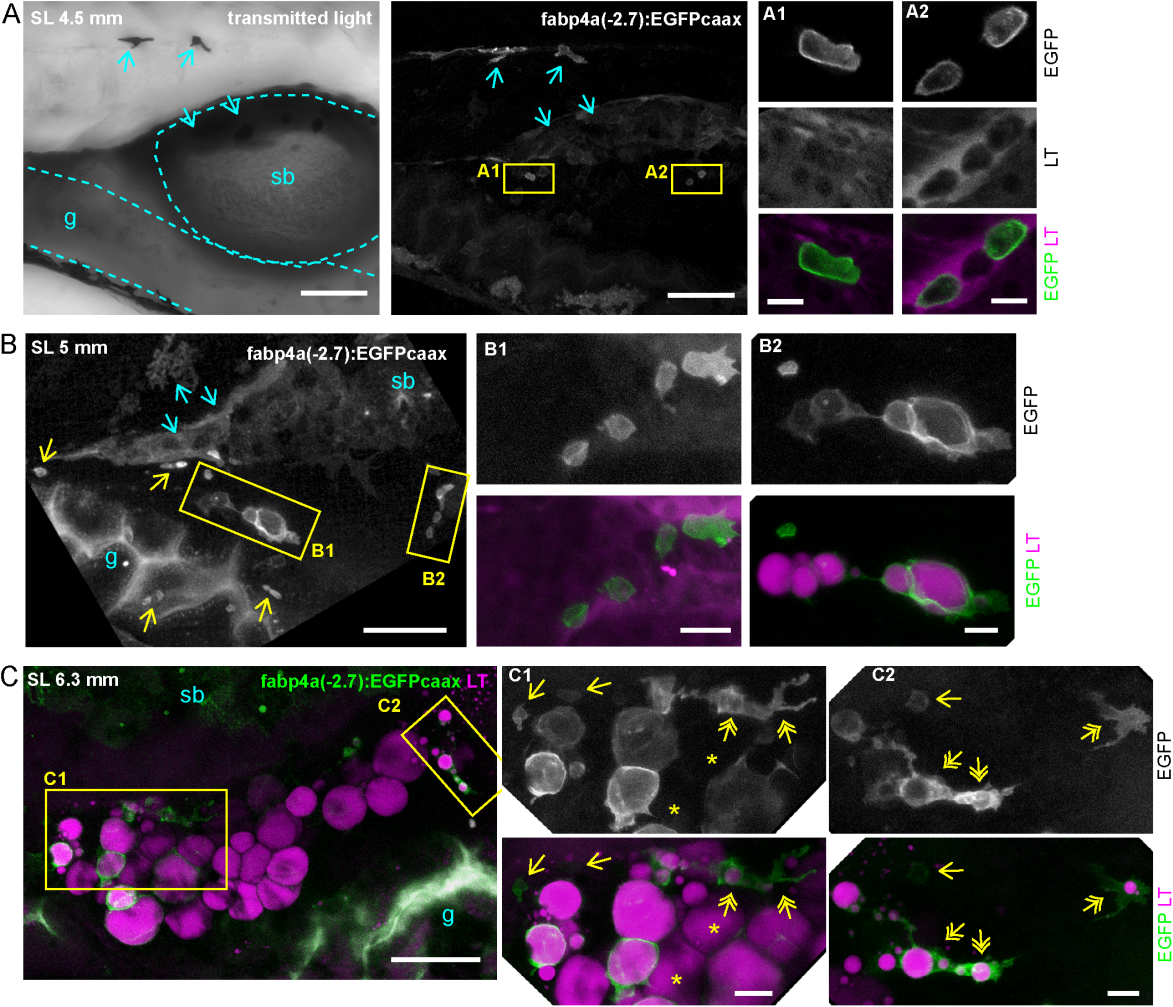
**Distribution of labeled cells in the abdominal region of live *fabp4a(-2.7):EGFPcaax* larva of different stages.** Larvae of the indicated stages were stained with LipidTOX-Red, mounted in agarose and imaged using confocal microscopy. (A) Transmitted light and 3D projection images of a larva of SL 4.5 mm (8 dpf). Yellow rectangles denote cells with transgene labeling. Insets A1 and A2 show confocal sections of these regions. Note the membrane localization of EGFP and the lack of LipidTOX-Red labeling. Cyan arrows indicate pigment cells expressing the transgene. (B) 3D projection images of a larva of SL 5 mm (12 dpf). Yellow rectangles denote EGFP+ cells, magnified in B1 and B2. Cells with lipid droplets as well as without them (yellow arrows) can be seen in the same larva in different positions. (C) 3D projection images of a larva of SL 6.3 mm (16 dpf) with initial PVAT depot formation. Note the presence of EGFP+ cells with unique cell-filling lipid droplets, irregular cells with several lipid droplets (yellow double arrows) and small cells without lipid droplets (yellow single arrows). Asterisks indicate cells without EGFP expression. sb, swim bladder; g, gut. Scale bars: A: 100 μm (panoramic view); 10 μm (insets); B: 100 μm (panoramic view); 20 μm (insets); C: 100 μm (panoramic view); 20 μm (insets).

Previously, it has been reported that lipid droplets are first evident at the right side of the abdomen of early larvae, in ventral and posterior positions with respect to the swim bladder (Flynn et al., 2009; Minchin and Rawls, 2017b). Thus, we turned our attention into that region in larval stages and stained lipid droplets with LipidTOX-Red. Interestingly, early larvae of standard length (SL) 4.5 mm (8 dpf) showed labeling of small cells within the abdomen. At higher magnification we observed the expected surface localization of EGFP. However, these cells did not present lipid droplets (Fig. 3A). We also observed labeling of cells within the trunk in dorsal positions which corresponded to the presence of pigment cells in transmitted light images. Other signals in the images corresponded to autofluorescence of gut contents in the ventral-most part of the larvae and to pigment cells in the dorsal half of the swim bladder (Fig. 3A). Despite taking several actions to diminish these confounding signals (16 h food restriction previous to imaging; incubation with epinephrine), they were persistent. Nevertheless, based on the surface localization and intensity of the EGFP signal it was possible to clearly distinguish these EGFPcaax positive and lipid droplet-free cells (EGFP+/LD−).

**Fig. 4.**
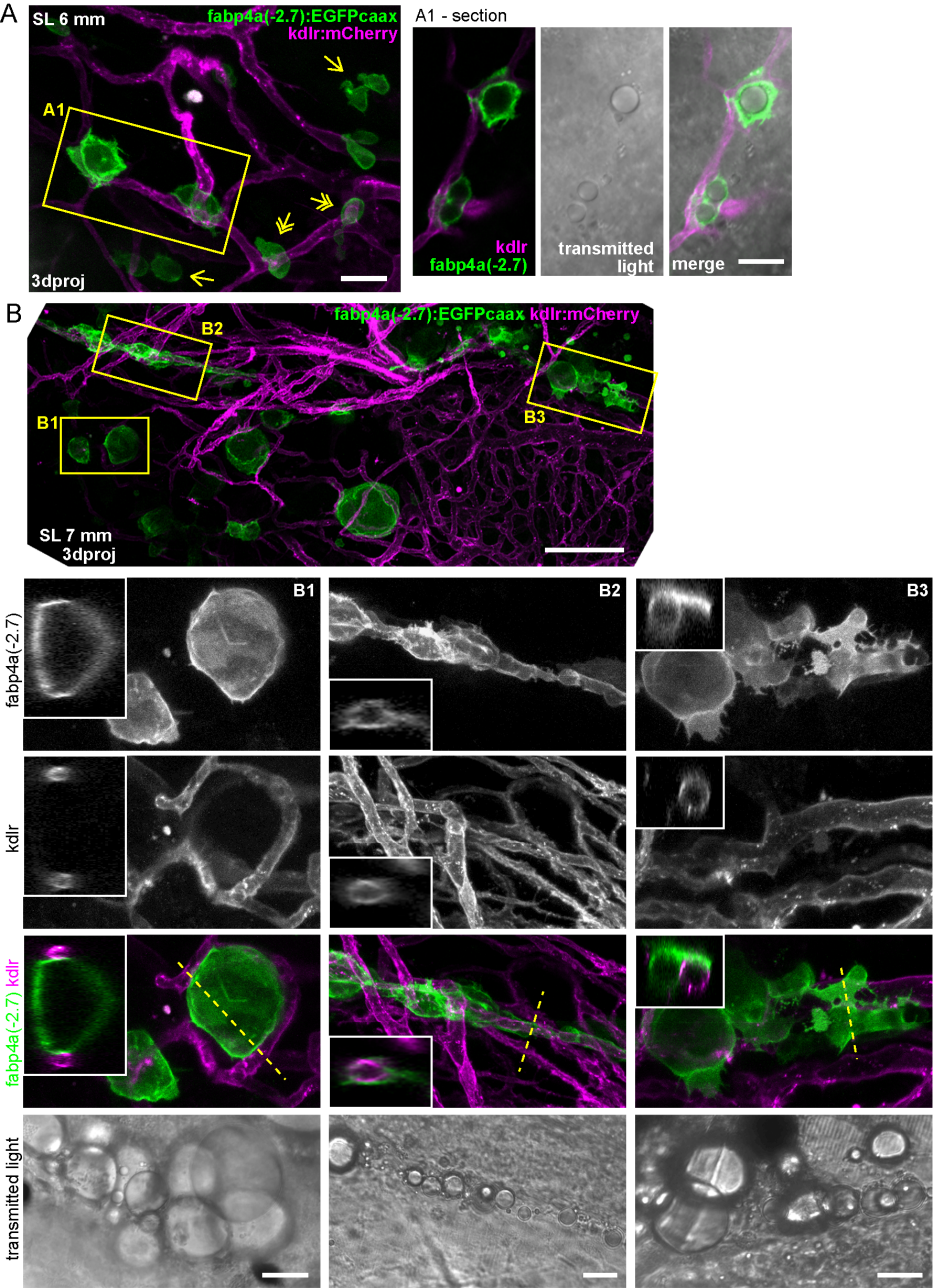
**Interaction of early adipocytes with blood vessels.** Live larvae from the cross of *fabp4a(-2.7):EGFPcaax* and *kdlr:mCherry* fish lines were imaged through confocal microscopy. Images presented here are 3D projections or single sections, as indicated. (A) Larva of SL 6 mm (13 dpf) with many EGFP+ cells in its abdominal area, a few of them having lipid droplets (inset A1). Some of the cells are in contact with blood vessels (double arrows) and some of them are not (single arrows). (B) Larva of SL 7 mm (16 dpf), with PVAT and AVAT depots (only some cells of each depot expresses EGFP). Insets B1, B2 and B3 show EGFP+ cells with lipid droplets in close apposition to blood vessels and in some cases surrounding them (B2). 3D projections and sections through the position indicated by the dashed line are shown. Scale bars: A: 100 μm; B: 100 μm (panoramic view), 20 μm (insets).

Next, we evaluated older larvae to assess whether these EGFP+ cells in the abdominal region could accumulate lipids. Importantly, larvae of SL 5 and 6.3 mm (12 and 16 dpf, respectively) clearly showed cells with surface EGFP signal and lipid droplets of various diameters (Fig. 3B,C). We also noted that some of these cells had irregular forms and projections (Fig. 3C, double arrows). Larvae of these ages also had rounder cells almost completely filled with one big lipid droplet (Fig. 3B,C). In mice, pre-adipocytes have been found to reside near blood vessels (Tang et al., 2008). Thus, we analyzed the relationship of early adipocytes with the vasculature by crossing *fabp4a(-2.7):EGFPcaax* fish with the *kdlr:mCherry* line, which labels endothelial cells. We observed EGFP+ cells both in close apposition and at some distance of vessels (Fig. 4A). Moreover, when cells with lipid accumulation were observed in the PVAT or AVAT depots, some usually appeared in close contact with vessels, sometimes with extensions surrounding them (Fig. 4B).

**Fig. 5.**
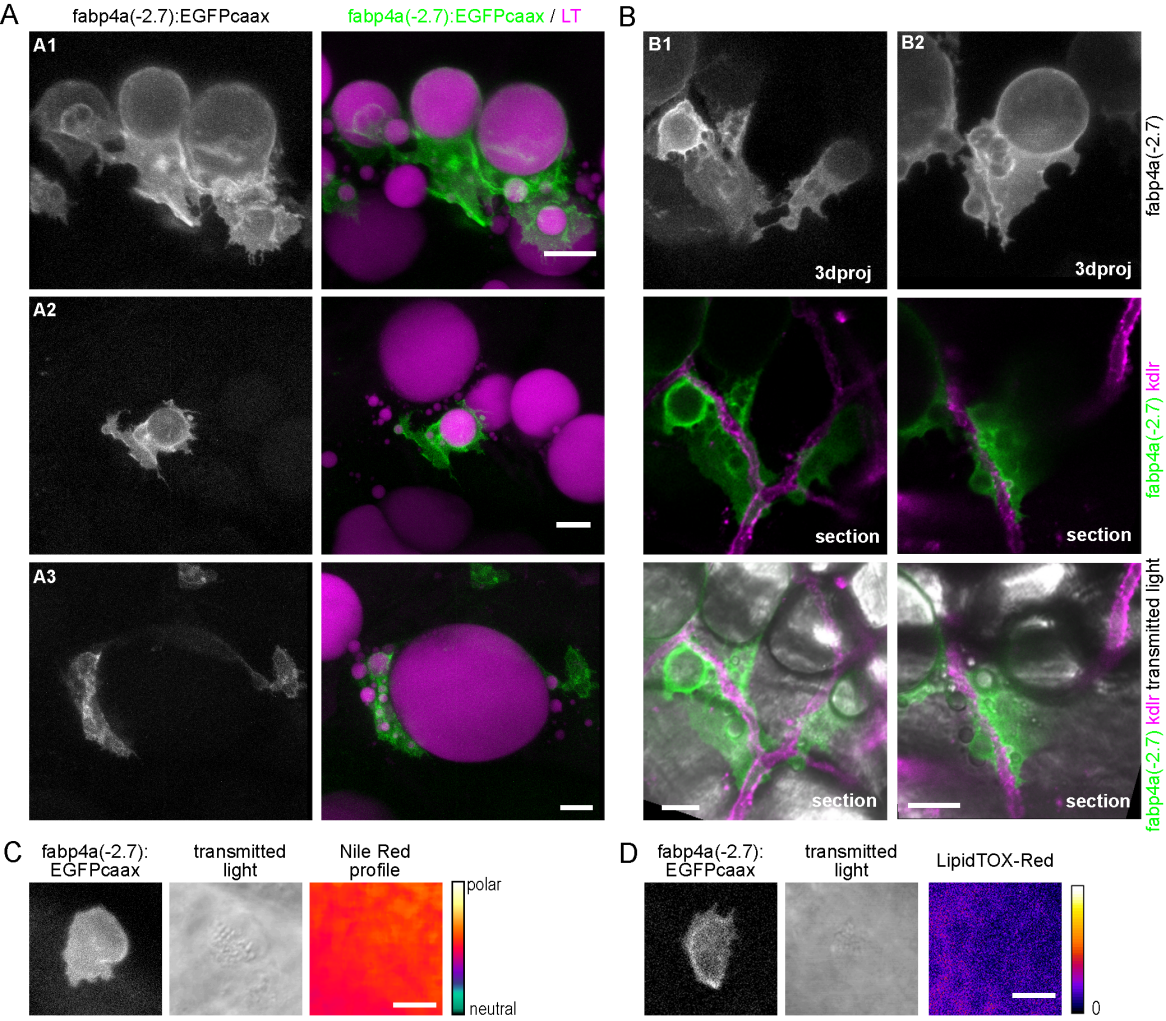
**Different cell morphologies observed in *fabp4a(-2.7):EGFPcaax* larvae.** (A) *fabp4a(-2.7):EGFPcaax* larvae of 21 dpf were labeled with LipidTOX-Red and imaged *in vivo* through confocal microscopy. Images are 3D projections of confocal stacks, to show different cell morphologies found in these larvae*.* (B) Images of *fabp4a(-2.7):EGFPcaax*; *kdlr:mCherry* larvae of SL 8 mm (19 dpf). Note labeled cells in the AVAT depot with cytoplasmic projections which lay in close apposition to blood vessels. Images are 3D projections or sections as indicated. (C,D) High magnification confocal sections of EGFP+ cells in larvae stained with Nile Red (C) or LipidTOX-Red (D). The cytoplasmic inclusions observed in transmitted light in these cells showed a polar lipid profile (evidenced by Nile Red staining, C) and low signal intensity of LipidTOX-Red (D). Scale bars: A: 20 μm; B: 20 μm; C,D: 10 μm.

We expanded our analysis until 21 dpf larvae (larvae of SL 7–8 mm) and observed EGFP+ positive cells with different morphologies (Fig. 5A,B). We observed rounded cells filled with a single lipid droplet, which likely correspond to mature adipocytes, in some cases having cell projections. Other cells, usually located at the periphery of the depots, typically showed one or more smaller lipid droplets and more irregular morphologies, also with membrane projections (Fig. 5A). In many occasions we observed cells in close proximity to vessels or with extensions surrounding them (Fig. 5B). Remarkably, in all stages analyzed in this work, EGFP+/LD− cells were present. These cells could be observed not only in the abdominal region in AVAT and PVAT but also surrounding the gut in different positions (Fig. 3B), including the cloaca region (Fig. S4). Interestingly, using transmitted light and high magnification we observed inclusions within EGFP+/LD− cells (Fig. 5C,D). We performed time lapse acquisitions of those cells for a short period of time. During these time lapse movies we observed that inclusions moved within the cytoplasm (Movie 1). Furthermore, we observed that cells could remain static or have directional movement over a cell diameter distance (Movie 1). This behavior was accompanied by the formation of protrusions that were also evident in our single time point observations (Figs 3B and 5C,D).

Altogether, our results show that *fabp4a(-2.7):EGFPcaax* expressing cells are present in different larval stages, from just before the beginning of the accumulation of fat to later stages where lipid depots are readily visible. Furthermore, we found labeled cells with lipid droplets of different sizes, confirming that cells expressing *fabp4a(-2.7):EGFPcaax* in the abdominal region of larvae are adipocytes in different stages of differentiation. Even though our fish line also expresses EGFP in pigment cells, *in vivo* 3D analysis of the abdominal region efficiently allowed us to distinguish early and mature adipocytes based on localization and cell shape. Furthermore, the results underscore a tight relationship between adipocytes and vessels during their differentiation, and the coexistence of EGFP+ lipid-filled cells with EGFP+/LD− cells in the tissue.

### Analysis of the lipid metabolic profile of early adipocytes with Nile Red fluorescence and spectral phasor plot analysis

As mentioned before, we hypothesized that EGFP+/LD− cells were in fact early adipocytes. Early adipocytes initiate lipid accumulation as part of their differentiation program, and thus would show a mixed lipid environment with neutral and polar components. The quantification of these components has been carried out before in cultured cells through Nile Red fluorescence analysis using spectral phasors (Di Giacinto et al., 2018; Maulucci et al., 2018). In our transgenic larvae, the fluorescence of EGFP could be used as a third component to identify the cells of interest (EGFP+ cells). Thus, we took advantage of the spectral phasor analysis to study the Nile Red spectral shift in the presence of EGFP fluorescence. A similar approach has been used to study membrane polarity using LAURDAN in the presence of mRuby fluorescence (Sameni et al., 2018). For the cellular lipid metabolic profile, wild-type larvae stained with Nile Red or *fabp4a(-2.7):EGFPcaax* larvae with or without staining with Nile Red were imaged using hyperspectral detection and the images were analyzed using the advantages of the model-free spectral phasors approach (Fig. S5, the analysis procedure is described in depth in the Material and Methods section) (Malacrida et al., 2017).

Wild-type larvae stained with Nile Red or *fabp4a(-2.7):EGFPcaax* larvae without staining were analyzed first to set the extremes of the distributions in the phasor plot (Fig. S5B). Notice that the Nile Red fluorescence was spread in a trajectory due to the heterogeneity in the polarity of Nile Red environments provided by the intracellular membranes. The position along the trajectory represents pixels corresponding to different fractions of membranes with more or less polarity. In the EGFP+ cells labeled with Nile Red, the linear combination for the Nile Red was dragged towards the EGFP position (Fig. S5C). Thus, the extremes of the Nile Red trajectory can be considered as two components and the EGFP as the third component. This strategy enabled us to generate masks for individual EGFP+ cells and to analyze their lipid polarity profile, avoiding the Nile Red signal from other cells. An example image is shown in Fig. S5C. Two cells, one with lipid droplets (cell A) and another without them (cell B), generated clusters at the phasor plot with unequivocally different distribution profiles. To analyze the lipid polarity profile on each of them, we obtained the polarity fractional plot (Fig. S5D). The analysis of cell A yielded a multimodal distribution with higher representation of intermediate zones, whereas cell B gave a single peak in the polar lipid region.

**Fig. 6.**
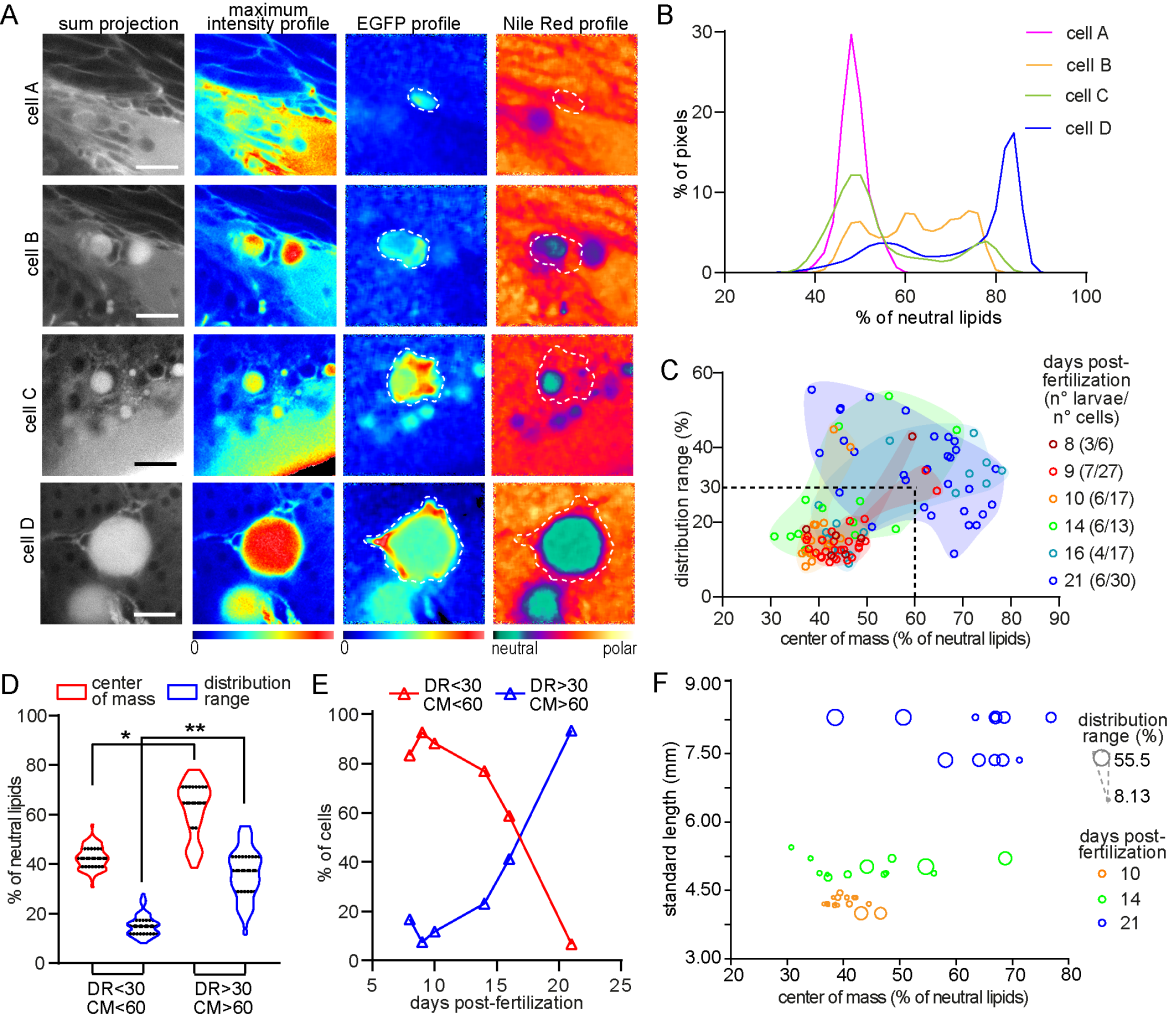
**Larvae in different stages present cells with distinct lipid metabolic profiles.** (A) Representative hyperspectral images of adipocytes (‘cell A’ to ‘cell D’) in different stages of differentiation. Raw images are presented in gray and intensity based color scale. Images generated after phasor plot analysis make evident the EGFP and Nile Red profiles which are represented separately by different color scales. Scale bars: A: 20 μm. (B) Plot with the normalized distribution of the image pixels with respect to the fraction (expressed as percentage) of neutral lipids for each cell in A. (C) Scatter plot showing the distribution range and center of mass of the cells analyzed in different larval stages. Both variables are expressed as the percentage of neutral lipids. The group of data coming from the same larval stage were enclosed by a manually generated convex hull shape just for visualization purposes. The dashed lines (DR=30; CM=60) generates the two regions considered in D and E. The numbers in brackets indicate the total number of larvae and the total number of cells analyzed in each stage. (D) Data within (DR<30/CM<60) and outside (DR>30/CM>60) the region considered in C were separated and compared. For CM: (*) *P*=2.06×10^−13^ for median comparison (Mann–Whitney test), *P*=1.62×10^−4^ for coefficient of variation comparison (Fligner-Killeen test); (**) *P*=2.24×10^−16^ for median comparison (Mann–Whitney test), *P*=7.1×10^−6^ (Fligner-Killeen test). (E) Representation of the percentage of cells in each group (DR<30/CM<60 and DR>30/CM>60) with respect to the larval stage presented in dpf. (F) Representation of the distribution range (as the size of the dots) and center of mass for the cells within some of the larva analyzed; each larva had a different standard length.

Using this approach, we analyzed EGFP+ cells in larvae at different stages. Representative examples of the observed profiles are shown in Fig. 6A and B. Seemingly mature adipocytes with a big lipid droplet showed a peak in Nile Red profile in regions corresponding to the accumulation of neutral lipids as expected (Fig. 6A,B, ‘cell D’). Interestingly, it was possible to observe a small peak towards longer wavelengths, representing polar lipid components in the same cells, such as the plasma membrane. This was corroborated by the localization of these pixels: the former were localized centrally and the latter surrounded the whole cell (Fig. 6A, see Nile Red profile of ‘cell D’). EGFP+/LD− cells usually showed distributions enriched in polar components (Fig. 6A,B, ‘cell A’). Nevertheless, we imaged cells with several peaks or flatter distributions, probably representing transitions between polar and neutral lipid environments (Fig. 6A,B, ‘cell B’ and ‘cell C’). To summarize and present all the observed profiles the center of mass (CM) and distribution range (RD) of the lipid polarity profiles were calculated and used as characteristics of each distribution for comparison purposes (Materials and Methods; Fig. S5D). Within the plot of center of mass versus distribution range (Fig. 6C) it was possible to separate a subgroup of cells with statistically distinct median (for the center of mass and distribution range) and variability (only for the center of mass) compared to those cells outside this region (Fig. 6C,D, dashed line). The low CM and low DR means that the cells within this group were constituted mostly by polar lipids. These cells represented over 50% of the cells analyzed from 8 to 16 dpf (Fig. 6E). The rest of the cells analyzed layed outside the low CM-low DR region due to increasing accumulation of neutral lipids, which extended the DR and biased the CM towards higher values. The percentage of cells analyzed that can be classified in this sub-group increased with larva age (Fig. 6E). Of note, we observed cells with distinct lipid polarity profiles among larvae of similar standard length and in some cases within the same larva (Fig. 6E). These observations implied the coexistence of adipocytes in different stages of differentiation within the same larva, suggesting that differentiation *in vivo* is continuous and asynchronous. These results indicate that our zebrafish *fabp4a(-2.7):EGFPcaax* transgenic line together with hyperspectral imaging and the spectral phasor analysis shown here is a powerful tool to study changes of the intracellular lipid environment in differentiating adipocytes in live zebrafish larvae.

## DISCUSSION

A number of studies have started to address the role of compounds on lipid metabolism and storage using zebrafish as a model system (Landgraf et al., 2017). Current knowledge on zebrafish adipose tissue is restricted to fat accumulation capacity using lipophilic dyes due to the lack of specific cell markers. Here we generated and analyzed a new zebrafish line to specifically label adipocytes along their differentiation *in vivo*.

### Expression pattern of *fabp4a(-2.7):EGFPcaax*

We generated a new transgenic line cloning the proximal part of the promoter of the lipid transporter gene *fabp4a* and used it to direct the expression of a membrane associated form of EGFP. It was shown previously that *fabp4a* is expressed in the lens, midbrain as well as in blood vessels in the head and trunk of 2 dpf embryos (Liu et al., 2007), an expression pattern that we also observed with our WMISH assay in 2 dpf embryos. However, the *in vivo* analysis of 2 and 5 dpf embryos showed that *fabp4a(-2.7):EGFPcaax* transgene is not expressed in either blood vessels, midbrain or lens cells. In the case of 15 dpf larvae, *fabp4a* mRNA was previously reported to be expressed in abdominal cells with or without neutral lipid accumulation as well as in trunk vessels (Flynn et al., 2009). In agreement with this report, our WMISH assay in larvae showed the expression of endogenous *fabp4a* in PVAT and AVAT depots as well as in blood vessels. However, the expression of *fabp4a(-2.7):EGFPcaax* transgene colocalized with endogenous expression of *fabp4a* only in cells of AVAT and PVAT depots. Thus, the expression pattern of *fabp4a(-2.7):EGFPcaax* recapitulates primarily the adipose tissue domain of the endogenous expression pattern of *fabp4a.* Interestingly, a recent report has shown that the 1.5 kb proximal region of *fabp4a* directing the expression of cytoplasmic EGFP, also results in an expression pattern restricted to adipose tissue (Mao et al., 2021).

Our *fabp4a(-2.7):EGFPcaax* transgene is expressed in pigment cells (melanophores and iridophores), a domain that does not coincide with the endogenous expression of *fabp4a*. The presence of this expression domain may be due to the action of enhancers or repressors present in the genomic region where the transgene was integrated. This is a common drawback of using random insertion of transposons for transgenesis and highlights the importance of using complementary strategies such as insulators or targeted transgene insertion (Caldovic et al., 1999; Roberts et al., 2014). Importantly, however, the extra domain of expression in pigment cells did not hinder the utility of the transgenic line as it can be clearly separated from the adipose-related signal by considering the relative position of cells in 3D images.

Another particular aspect of this new transgenic fish line is that the level of expression of EGFP varies among cells within each larva. This variability remained even after three outcrosses with the wild-type fish line, discarding a mosaicism-based effect. Thus, variation among cells may reflect different cellular states along differentiation or, alternatively different subtypes of adipocytes. Progenitor and adipocyte subtypes with differential gene expression profiles within individual depots have been reported previously in mice and humans, probably reflecting their distinct developmental origins (revised in Luong et al., 2019). The promoter region used to generate the present transgenic line includes several binding sites for Pparγ and NF-Kβ p50 that were able to modulate transcription in reporter assays (Laprairie et al., 2017). Moreover, Pparγ has been shown to bind the *fabp4* promoter in a brown adipocyte cell line (Tontonoz et al., 1994). Also, it has been reported that *fabp4* is regulated by VEGFA-DLL4/NOTCH and insulin-FOXO1 pathways in endothelial HUVEC cells (Harjes et al., 2014).

In humans, plasma levels of Fabp4, which is mainly produced by adipocytes, have been positively correlated with cardio-vascular disease, type-II diabetes and also with the progression of other diseases by a still undefined mechanism (Prentice et al., 2019). Thus, it would be interesting to analyze which factors contribute to the expression levels of *fabp4* as they may be of clinical relevance, and our results showing significant variability among adipocytes suggests this zebrafish line could be useful to this end.

### Characterization of early adipocytes and their relationship to blood vessels

As mentioned, our detailed microscopic analysis of *fabp4a(-2.7):EGFPcaax* larvae showed labeled cells with different characteristics. In the abdominal region, where WAT depots form first, we observed both EGFP+ cells with lipid droplets of various sizes and others without them. These observations are in agreement with previous reports showing that *fabp4a* is expressed in cells with and without lipid droplets in embryonic stages and early larvae (Flynn et al., 2009). Accordingly, we found EGFP+ cells in the ventral region of the tail and EGFP+/LD− cells in the abdominal region of early larvae (8–10 dpf) well before the initiation of fat accumulation. EGFP+/LD− cells were also observed in older larvae, coexisting with mature adipocytes in fat depots. Our analysis of Nile Red emission of EGFP+/LD− cells in the abdominal region revealed polar lipid or intermediate profiles. Thus, our results indicate that the *fabp4a(-2.7):EGFPcaax* transgene labels adipocytes in the PVAT and AVAT depots ranging from early stages of differentiation to mature differentiated cells. Future work will be focused towards analyzing the identity (pre-adipocyte versus early adipocyte) of EGFP+/LD− cells.

EGFP+/LD− cells in the abdominal region presented inclusions when observed with transmitted light at high magnification. These inclusions were LipidTOX-negative or showed a polar lipid profile in Nile Red hyperspectral analysis, and were highly motile within the cell when analyzed using time lapse acquisitions. Further experiments are required to determine the nature of these inclusions. One interesting possibility is that they may represent initial stages of lipid droplet formation in which the amount of accumulated neutral lipids is not enough to be observed through LipidTOX labeling. LD are formed through accumulation of neutral lipids within the lipid bilayer of the ER, initially forming structures denominated lenses which grow and bud becoming lipid droplets (Olzmann and Carvalho, 2019). Genetic labeling tools that have been developed to evidence initial neutral lipid accumulations may be implemented to study the conservation of early lipid droplet formation mechanisms in zebrafish (Kassan et al., 2013; Wang et al., 2016).

WAT progenitors expressing PPARγ have been reported to reside in the mural compartment of adipose blood vessels in mice (Hilgendorf et al., 2019; Tang et al., 2008). As an analogy to mammals, some authors have hypothesized that WAT progenitors in zebrafish may derive from perivascular pre-adipocytes or, alternatively, from hematopoietic tissue located in the caudal region (Salmerón, 2018). In double-labeled larvae, we found EGFP+/LD− cells both in contact and at a distance of blood vessels. In contrast, all EGFP+ cells with lipid droplets were observed in contact with blood vessels. EGFP+/LD− cells were also present surrounding the gut at different positions along the antero-posterior axis. Furthermore, our time lapse acquisitions revealed that these cells had the capacity to migrate. Thus, our results are consistent with the previous formulated hypothesis and *in vivo* time lapse microscopy of EGFP+ cells combined with cell tracing may provide further information. For this, new methods to maintain larvae alive through extended periods of time will be needed, since in our hands, larvae remained alive only for a few hours after mounting in agarose.

Our work also provides information about adipocytes during differentiation and in their mature state. As our transgenic approach included a membrane associated form of EGFP, we could clearly identify the presence of membrane protrusions in early and mature adipocytes. In double-labeled larvae we could appreciate that these membrane protrusions reached blood vessels, suggesting the presence of physical connections. Whether this interaction is direct between cell membranes or indirect through ECM components remains to be determined. Evidence exists in favor of the latter hypothesis, showing that modification of extracellular matrix composition by endothelial cells directs the hyperplasia or hypertrophy of adipocytes (Minchin et al., 2015). Alternatively, extensive evidence supports that several soluble factors coordinate adipogenesis and angiogenesis in obesity as well as in adipose-derived stem cell therapy (Hutchings et al., 2020; Lemoine et al., 2013). Furthermore, secretion of factors by peri-arterial adipocytes can mediate protection or inflammation of the adventitia and atherosclerosis development (Kim et al., 2020). Much less information is available on the interaction of adipocytes and vessels during formation of the adipose tissue (Cao, 2007). Our results suggest an intimate relationship of early adipocytes with blood vessels, probably through cell surface molecules. We hypothesize that these interactions may be instrumental in the acquisition of lipids from blood vessels as well as in regulating adipose depot growth.

### Nile Red and phasor approach to characterize *in vivo* cell lipid metabolism

We used the new *fabp4a(-2.7):EGFPcaax* line to implement a tool for *in vivo* analysis of lipid environment using Nile Red hyperspectral imaging and its analysis through spectral phasor plots (Maulucci et al., 2018). Early adipocytes initiate lipid accumulation as part of their differentiation program, and thus would show a mixed lipid environment with neutral and polar components in their profile. Indeed, as mentioned before, EGFP+/LD− cells showed different profiles, ranging from polar-lipid environments to intermediate polarity-lipid environments. Several groups have studied the lipid composition of *in vitro* differentiating adipocytes of different origins through disruptive methods (Miehle et al., 2020). For example, human undifferentiated adipocytes were enriched in membrane phospholipids such as phosphatidylethanolamines, phosphatidylcholines and sphingomyelins. Meanwhile, completely differentiated cells were shown to present diacylglycerols, lysophosphatidylethanolamines and triacylglycerols in addition to membrane phospholipids. Thus our results are consistent with previous analyses, and importantly, provide a base to build on the metabolic analysis of individual cells in their natural context.

Our data show that the technique is sensitive enough to detect lipid environment changes in a non-invasive way and for a specific cell identity, opening the possibility of using this tool to evaluate the progression of differentiation *in vivo* or the effect of drugs on lipid metabolism or genetic interventions. Future development of other fish lines using earlier molecular markers will improve the observation of cells in different stages. For example, work in mice have used *pref1* and *zfp423* to mark adipose tissue progenitors and pre-adipocytes (Gupta et al., 2010; Hudak et al., 2014). Both of these genes are present in zebrafish and may be useful to track the origin of the adipocyte lineage.

## Conclusion

In this work we introduced a new zebrafish line labeling adipocytes from early stages up to fully differentiated cells. Furthermore, we described the interaction of early and differentiated adipocytes with blood vessels and evidenced early lipid metabolic changes *in vivo*. We anticipate that the new transgenic line described here will be a useful tool to study the cell biology of adipocytes in the context of the tissue and the whole organism, their interaction with blood vessels and their differentiation *in vivo*. Recently, new fish lines labeling lipid droplets have been generated (Lumaquin et al., 2021; Wilson et al., 2021 preprint) that may be combined with the *fabp4a(-2.7):EGFPcaax* line presented here for screening approaches focused on genetic and environmental factors affecting early adipocyte differentiation. The *fabp4a(-2.7):EGFPcaax* fish lines and the genetic tools available in zebrafish, combined with two-photon and multiplexing microscopy will surely provide a powerful platform to gain in depth information on adipogenesis and its *in vivo* determinants.

## MATERIALS AND METHODS

### Zebrafish maintenance and growth

We worked with AB/TU (wild-type fish line), Tg(*kdlr:mCherry*)is5 (blood vessel labeling; Wang et al., 2010) and Tg(*fli1:EGFP*)y1 (blood vessel labeling; Lawson and Weinstein, 2002). *Danio rerio* adults were maintained in a stand-alone system (Tecniplast), at 28°C, 800 µS/cm^2^, and pH 7.5, with a diet based on live 48 h-post eclosion *Artemia salina* (artemia cyst from Artemia International) and pellet (TetraMin, tropical flakes, Tetra). Embryos were raised in Petri dishes with aquarium water at 28.5°C (50 larvae per 10 cm Petri dish) and bleached at 24 h post-fertilization. For growth of larvae we used Larval AP100-1 (<50 μm; Zeigler) from 5 to 30 dpf and Golden Pearl Reef & Larval Diet (100–200 μm; Brine Shrimp Direct) from 15 to 30 dpf. Dry food were administered twice per day plus one extra feed of live 24 h-post hatching *A. salina.*

Embryonic staging was performed according to Kimmel (Kimmel et al., 1995) up to 5 dpf and larvae staging (after 5 dpf) was done according to Parichy (Parichy et al., 2009). SL is the distance between the tip of the nose and the caudal peduncle, and it correlates linearly with the growth of adipose tissue as well as the development of other characteristics in larval zebrafish (Minchin and Rawls, 2017b). All protocols (n° 007-19, 009-19, 010-19, 011-19) were approved by the Institut Pasteur de Montevideo ethics committee for the use of animal models (CEUA) and performed by trained, certified staff.

### Promoter cloning

For identification of the potential promoter regions we combined manual analysis and a trial version of Gene2Promoter software (Genomatix). We then designed primers using the Primer-Blast tool from NCBI (Table 1). Candidate primers were blasted against the whole zebrafish genome using the BLAT tool from UCSC Genome Browser. Restriction sites were added at their 5′end to enable directional cloning (underlined in Table 1).

**Table 1.**
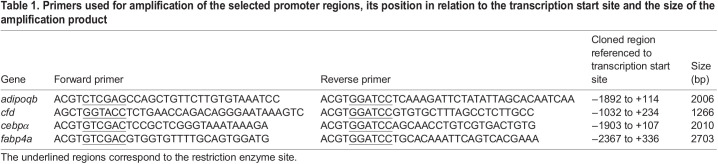
Primers used for amplification of the selected promoter regions, its position in relation to the transcription start site and the size of the amplification product

We used zebrafish high-molecular weight genomic DNA, extracted as previously described (Sambrook et al., 1990) from 48 hpf TAB embryos. For *fabp4a*, the BAC DKEY-241P5 (Source BioScience) was used as a template. Each region was first cloned into pCRII plasmid using TOPO-TA Cloning Kit (Thermo Fisher Scientific). After sequencing, they were sub-cloned into p5′Entry-MCS plasmid from Tol2 Kit (Kwan et al., 2007) through digestion and ligation with T4 ligase (Thermo Fisher Scientific). We recombined each p5′Entry vector with pMiddle Entry vector coding for EGFPcaax (caax is a prenylation signal, directing EGFP to the plasma membrane), p3′Entry Vector with poly-A signal and the pDestTol2-CG2 backbone (with cardiac myosin light chain promoter directing the expression of GFP, *cmlc2:GFP*; and tol2 sites for insertion into the genome).

### Transgenic line generation

TAB5 embryos at one cell stage were injected with 10–20 pg of the desired vector plus 10–20 pg of Tol2 Transposase mRNA. We then selected 24 hpf embryos showing GFP fluorescence in the heart. We grew these embryos until 15–21 dpf when we analyzed the presence of fluorescence in adipose tissue. Selected individuals were outcrossed with the wild-type line until the third generation.

### Fixation, permeabilization and immunolabeling

To decrease pigmentation, embryos were treated with PTU 0.3% starting at 8 hpf. For the same purpose larvae were anesthetized using tricaine 0.04 g/l, incubated in epinephrine 10 mg/ml plus tricaine 0.04 g/l, mounted in methylcellulose and observed using the stereomicroscope to select larvae expressing GFP. Fixation was carried out in 4% PFA in PBS overnight at 4°C.

Fixed embryos and larvae were permeabilized and immunolabeled following the protocol described by Inoue and Wittbrodt with minor modifications (Inoue and Wittbrodt, 2011). Briefly, all steps were carried out at room temperature with agitation unless stated otherwise. Fixed embryos and larvae were washed in PBS plus 1% Triton X100 (PBST) (3×10 min), dehydrated in a methanol series (50:50 and 100:0 methanol:PBST) (1×10 min each) and incubated in 100% methanol at −20°C for 20 min. After rehydration in the same methanol series, we performed an antigen retrieval step with 150 mM Tris-HCl pH 9 (5 min at RT and 15 min at 70°C). After a wash step in PBST (10 min) and two washes in distilled water (5 min each) we further permeabilized samples incubating them in 100% acetone at −20°C for 20 min. Finally we washed the samples in PBST several times (6×5 min each). For immunofluorescence on WMISH embryos and larvae we followed the same protocol without the acetone permeabilization step.

For immunolabeling all steps were performed with agitation. We incubated permeabilized embryos and larvae in the blocking buffer (10% FBS plus 1% BSA in PBST) for 1 h at room temperature (RT). Primary and secondary antibodies were diluted in the incubation buffer (1% FBS plus 1% BSA in PBST). Antibody incubations were performed at 4°C for 3 days, and washes at RT with PBST. The antibodies used in this work were: anti-GFP (Invitrogen #A11122, 1/500), anti-rabbit-633 (Invitrogen #A21070, 1/1000).

### WMISH

We cloned a region of *fabp4a* previously used for probe generation (Flynn et al., 2009) using the following primers: fwd: GATCAAATCTCAATTTACAGCTGTTG; rv: TTCAAAGCACCATAAAGACTGATAAT and oligodT retro-transcribed cDNA as a template. The amplified region was ligated into pGEM T-easy vector (Thermo Fisher Scientific). Selected clones were checked through digestion and sequencing. The selected clone has the region 195 to 648 from *fabp4a* mRNA sequence, spanning the 3′ half of the CDS and part of the 3′UTR, flanked by T7 and SP6 promoters in 5′ and 3′, respectively. To synthesize the probes we amplified the template using T7 and SP6 primers and afterwards generated digoxigenin (DIG) labeled probes by *in vitro* transcription with T7 or SP6 polymerases, using Digoxigenin-11-UTP (Merck). As an additional specificity control we used a *slit2* antisense probe which has already been tested (generously provided by C. Davison, Facultad de Ciencias, Universidad de la República; Davison and Zolessi, 2021).

The WMISH technique was performed as previously described (Koziol et al., 2014) with modifications following Flynn et al. and Elizondo et al. (Elizondo et al., 2005; Flynn et al., 2009). A detailed protocol is available upon request. Briefly, embryos and larvae were fixed in 4% PFA prepared in PBS-DEPC water overnight at 4°C. PFA was then replaced twice with 100% methanol and samples were stored at −20°C until used. After rehydration in an ethanol series, larvae were permeabilized with 15 µg/ml Proteinase K (Fermentas) in PBS-0.1% Tween-20 (PBS-T) for 10 min (for embryos) or 30 min (for larvae) at RT. After a rinse with triethanolamine buffer (0.1 M, pH 8), they were treated twice with acetic anhydride (0.25% v/v for 5 min each), washed with PBS-T, refixed with 4% PFA in PBS-T for 20 min and washed extensively with PBS-T at RT. Pre-hybridization was performed overnight at 60°C in hybridization buffer (50% formamide, 5X SSC, 1 mg/ml Torula RNA, 100 µg/ml heparin, 1x Denhardt's solution, 0.1% Tween-20, 0.1% CHAPS, DEPC treated water). DIG-labeled probes were denatured at 80°C for 3 min and diluted to 0.2 ng/µl in the hybridization buffer. Hybridization was performed at 58°C for 2 days with agitation. Washing steps were done in hybridization buffer at 58°C with agitation, twice for 10 min each, then three times with 2X SSC plus 0.1% Tween-20 at 58°C for 20 min each, three times with 0.2X SSC plus 0.1% Tween-20 at 58°C for 30 min each, and finally twice with maleic acid buffer (MAB) at RT for 15 min. Samples were then blocked overnight at 4°C in 1% blocking reagent (Roche) plus 5% sheep serum in MAB and incubated with anti-DIG conjugated to Peroxidase (1/50; Merck #11207733910 Roche) in 1% blocking reagent diluted in MAB for 3 days at 4°C. Washing steps were done in MAB (three washes of 5 min, followed by three washes of 1 h). Fluorophore deposition was carried out with fluorescein-tyramide, prepared and developed as described by Hopman et al. (1998). After washes in PBS-T, samples were stored in 80% glycerol in PBS at −20°C until used.

### *In vivo* labeling and imaging

For *in vivo* lipid labeling, selected larvae were incubated in a 10 cm Petri dish (when labeled in group) or 12-well plate (when labeled individually) containing the lipophilic dye diluted in system water. We incubated the larvae with LipidTox Red (Invitrogen, 1/5000) for 1 h at 28°C or Nile Red (Sigma-Aldrich, 0.78 μM for adipose area quantification and 0.078 μM for emission spectra analysis) for 1 h at 28°C. Labeled individuals were anesthetized and incubated in epinephrine as described above and mounted in 0.8% low melting point agarose in a 3.5 mm glass bottom Petri dish. After solidification, the sample was covered with tricaine 0.04 g/l in system water. To ensure viability during the observation period, a block of agarose covering the region of the gills and the lower jaw was removed using a needle.

Low magnification images were acquired using a stereoscope (Nikon SMZ-445) with a canon EOS T3i-Rebel camera. More detailed *in vivo* images were acquired using epifluorescence or confocal microscopy. For epifluorescence we used an Olympus IX81 with 10x UPlan FLN 0.3 NA and 20x UPlan FLN 0.5 NA Olympus objectives. Confocal microscopy images were acquired with either a Zeiss LSM 800 or Zeiss LSM 880 with a 25x LD LCI Plan-Apochromat 0.8 NA Imm Corr DIC M27 (glycerol, oil, water, silicone) Zeiss objective. Hyperspectral imaging of Nile Red fluorescence was done using the lambda module in the Zeiss LSM 880 with the 25x objective, excitation the 488 Argon laser line was used and the spectra acquisition involved 22 step with 10 nm bandwidth (from 493 nm to 713 nm) using a PMT-GaAsP detector.

### Image analysis

Brightness-contrast adjustments were done using Fiji software (Schindelin et al., 2012). Fluorescence profile of MWISH images were generated using the Intensity Toolset from Imperial College of London FILM facility (https://www.imperial.ac.uk/medicine/facility-for-imaging-by-light-microscopy/software/fiji/).

For Nile Red hyperspectral data analysis we used the spectra phasor approach using Globals for Images SimFCS 4 software (G-SOFT Inc., Champaign, IL, USA). This method transforms the spectral data in each pixel to the real and imaginary component of the Fourier transform, as described earlier by Malacrida et al. (2016):(1)
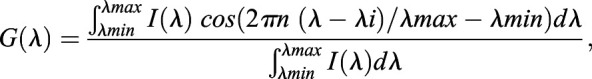
(2)
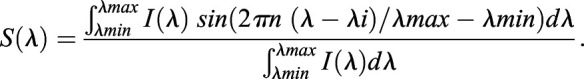
I(*λ*) is the intensity at each step, *n* the harmonic number and *λ_i_* is the initial wavelength. Each pixel in the image will be located at a single (G, S) position at the spectral phasor plot, yielding a cluster of points due to all pixels in an image. This transformation does not modify the original data and does not involve any fitting or any assumption of components. The position at the phasor depends on the spectrum maximum (phase angle, Θ) and the full width at half maximum (Modulation, M) (Fig. S5A), as:(3)
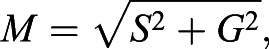
(4)
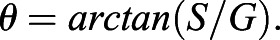
While red spectral shift implies increasing phase angle, the band narrowing moves the position toward the spectral phasor perimeter (modulation increases).

The spectral phasor plot enables the use of vector properties, such as the linear combination and the reciprocity principle. The linear combination allows the quantification of multiple components in a mixture as a sum fractions of single emitters. In our experiments, Nile Red presented complex photophysics that involved the emission from polar and neutral environments (membrane and lipid droplets, respectively). Furthermore, our phasor plots had an extra component from expression of EGFP. Using the three-component analysis developed by Ranjit and collaborators, we decomposed the fraction of Nile Red in the pixels with EGFP signal (Ranjit et al., 2019). We defined two individual cursor positions (two of the vertices) from the Nile Red trajectory extremes using images from wild-type larvae labeled with Nile Red, and the third position using images of unlabeled *fabp4a(-2.7):EGFPcaax* larvae. The reciprocity principle enables to trace back a region of interest from the spectral phasor (imaginary space) to the original image (real space; the opposite, from a segmentation in the real image to the phasor plot, is also possible). Using this property, we segmented individual cells selecting the corresponding pixels in the phasor plot. Then, we obtained the fractional contributions for the Nile Red trajectory as explained in detail elsewhere (Ranjit et al., 2019). For comparison purposes between different treatments we used the CM for the Nile Red fraction histogram as a central tendency value and the range of the distribution as a dispersion value. The CM for the distribution of each cell Nile Red fraction was calculated as:(5)
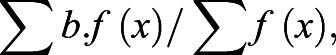
with ‘*b*’ being the percentage of pixels at the particular fraction ‘*f(x)*’ of the component ‘*x*’ (Malacrida and Gratton, 2018). The range of the distribution or distribution range (DR) was considered as the *f(x)* interval that contains 96% of the pixels. For its calculation we used the accumulated distribution for *f(x)* and determined the difference between the *f(x)* values corresponding to 2% and 98% of the accumulated distribution.

### Statistical analysis

The statistical analysis was performed using PAST software (Hammer et al., 2001) or Real Statistics Resource Pack software [Release 7.6, Copyright (2013–2021), Charles Zaiontz, www.real-statistics.com, accessed on March 2021]. For group comparisons we analyzed normality using Shapiro-Wilk test and homogeneity of variances using Levene test. Non-normal and homoscedastic distributions were compared with non-parametric tests (Kruskal–Wallis or Mann–Whitney with Bonferroni correction) as indicated in each case. Non-normal and heteroscedastic samples were rank transformed (Conover and Iman, 1981) and compared using Welch test and Games-Howell post-hoc test. For the comparison of coefficient of variation we used the Fligner-Killeen test.

## Supplementary Material

Supplementary information
