# Supplementary material for: Insights into in vivo adipocyte differentiation through cell-specific labeling in zebrafish
Source: Biol Open. 2021 Sep 9;10(9):bio058734. doi: 10.1242/bio.058734 (PMC8443861; doi:10.1242/bio.058734)
Supplement: Supplementary information [file biolopen-10-058734-s1.pdf]

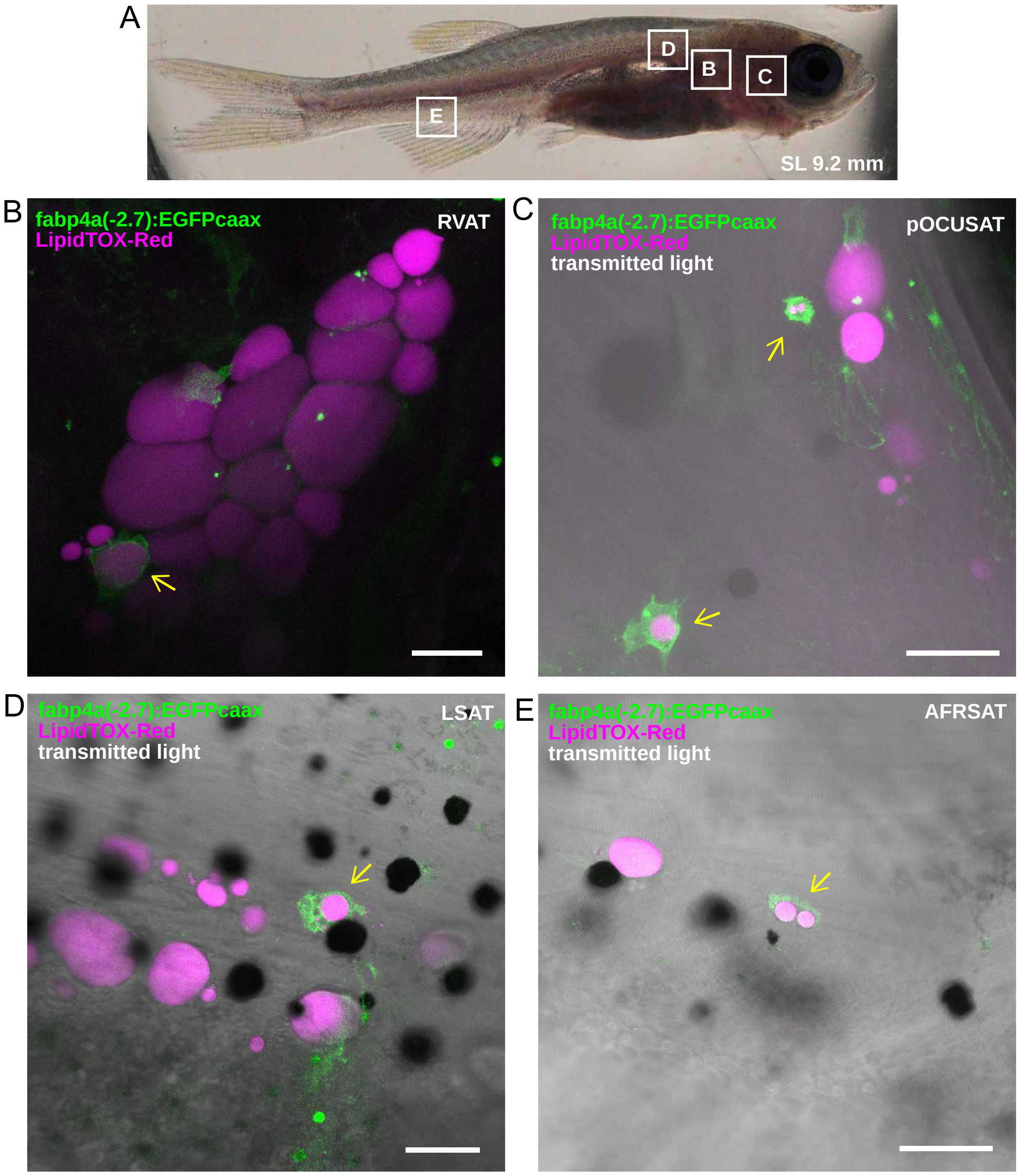

Fig. S1. Expression of *fabp4a(-2.7):EGFPcaax* in subcutaneous and visceral depots.

A. Low magnification image of a *fabp4a(-2.7):EGFPcaax* larvae at 27 dpf. Insets indicate the regions observed in B to E.

B-E. Confocal microscopy images of different adipose depots in live *fabp4a(-2.7):EGFPcaax* larvae labeled with LipidTOX-Red. Arrows indicate EGFP+ positive cells. RVAT: renal visceral adipose tissue; pOCUSAT: posterior ocular subcutaneous adipose tissue; LSAT: lateral subcutaneous adipose tissue; AFRSAT: anal fin ray subcutaneous adipose tissue.

Scale bars B-E: 50 µm.

Biology Open • Supplementary information

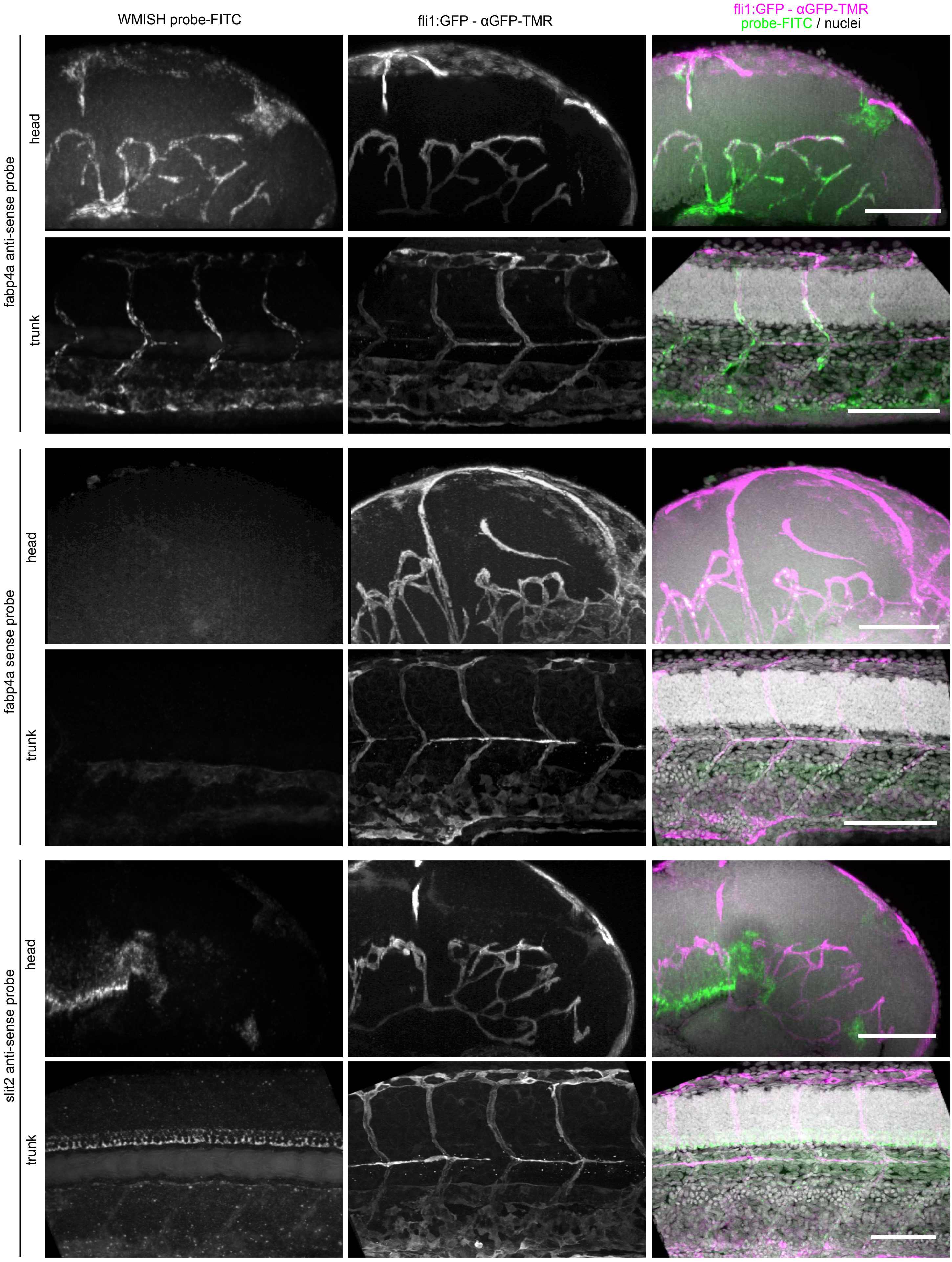

Biology Open • Supplementary information

Fig. S2. Set up of MWISH and immunofluorescence in embryos.

Images of 2 dpf *fli1:EGFP* embryos. WMISH was performed with antisense and sense probes against *fabp4a*, and with previously validated antisense probes for *slit2*. After immunolabeling with anti-GFP, embryos were imaged *in toto* through confocal microscopy. 3D projections generated from confocal stacks are shown. Scale bars: 100 μm.

Biology Open • Supplementary information

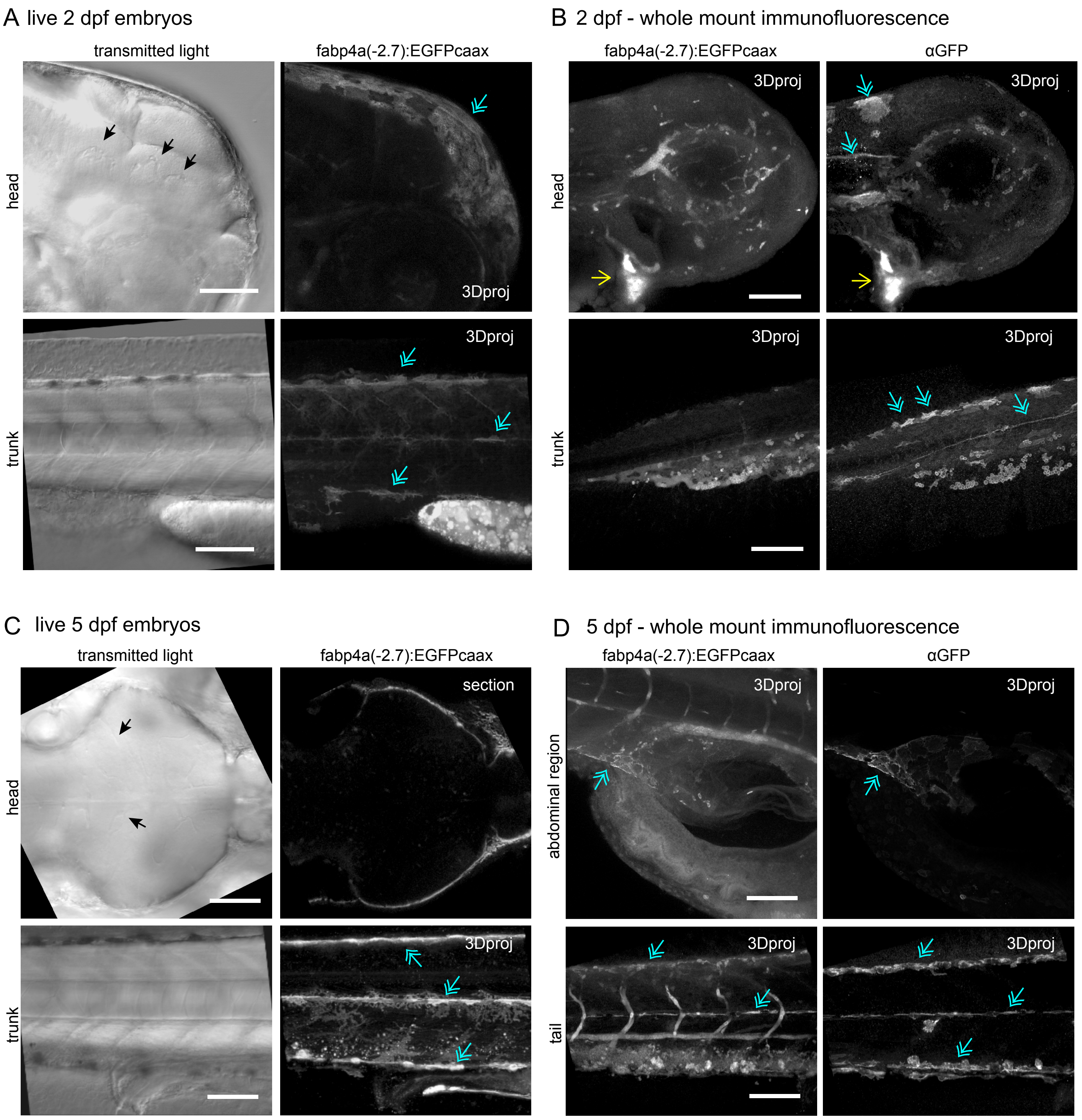
A live 2 dpf embryos
transmitted light
fabp4a(-2.7):EGFPcaax
head
trunk
3Dproj
B 2 dpf - whole mount immunofluorescence
fabp4a(-2.7):EGFPcaax
αGFP
3Dproj
C live 5 dpf embryos
transmitted light
fabp4a(-2.7):EGFPcaax
section
D 5 dpf - whole mount immunofluorescence
abdominal region
tail

Fig. S3. Expression domains of *fabp4a(-2.7):EGFPcaax* in embryos.

A and C. Images of live embryos of 2 and 5 dpf. Transmitted light or fluorescence images were acquired through confocal microscopy and presented either as single sections or 3D projections (3Dproj). In transmitted light it is possible to observe blood vessels (black arrows) and the lack of fluorescence colocalization.

B and D. Images of fixed embryos of 2 and 5 dpf immunostained with anti-GFP antibody. Both endogenous GFP and immunolabeling signals are shown. Yellow arrows show the positive immunolabeling signal in heart cells. Blue-double arrows indicate the presence of fluorescence in pigment cells, both in live and fixed embryos, as well as through immunolabeling.

Scale bars: A-D: 100 µm.

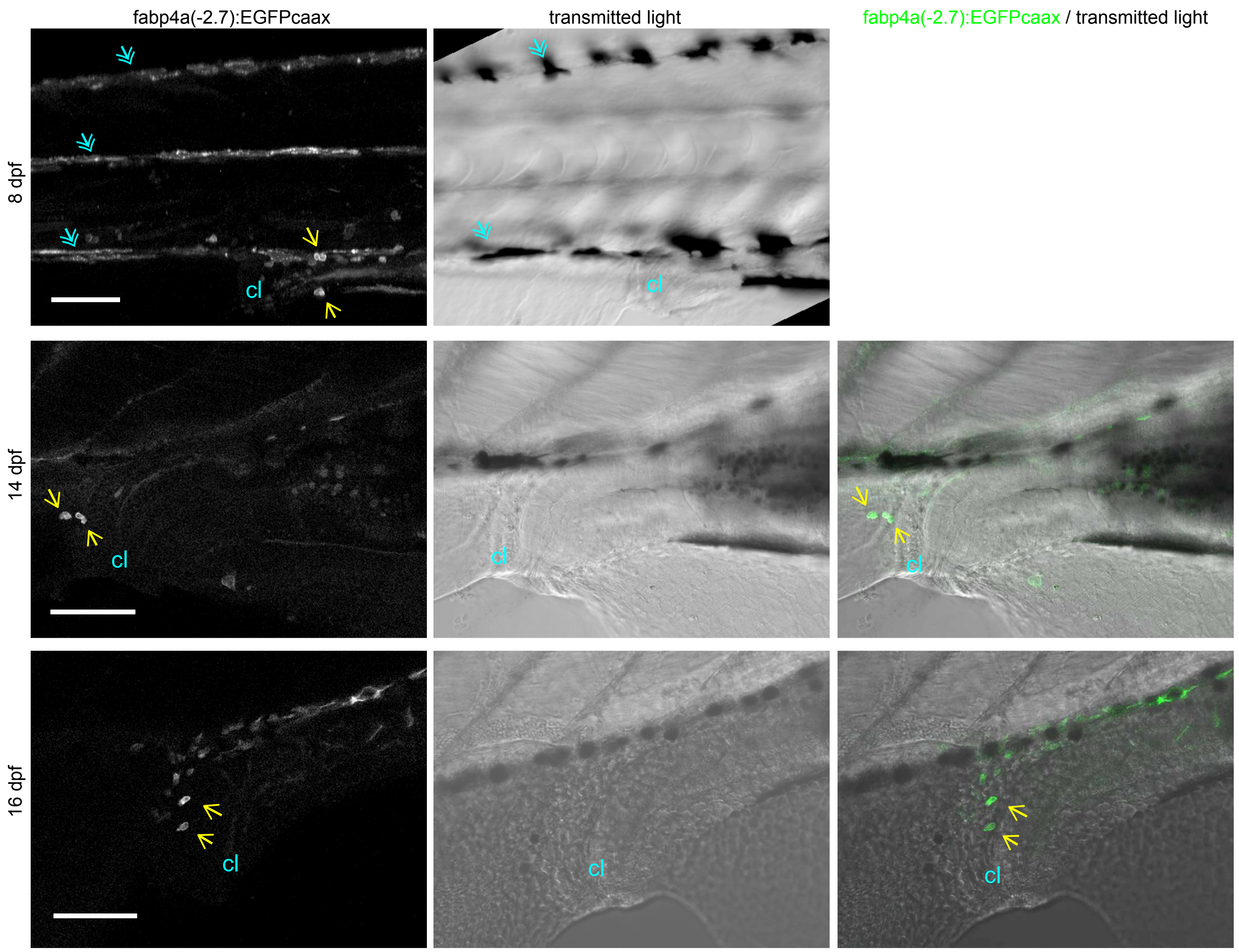

Fig. S4. Cells expressing *fabp4a(-2.7):EGFPcaax* in the tail region of larvae at different stages. Images of live larvae analyzed through confocal microscopy at different stages (expressed as dpf). EGFP+/LD- cells were present in all stages analyzed, including in the cloaca region (yellow arrows). The fluorescence image of 8 dpf larvae is a 3D projection to note the presence of labeled pigmentary cells (cyan double arrows). cl: cloaca. Scale bars: 100 µm.

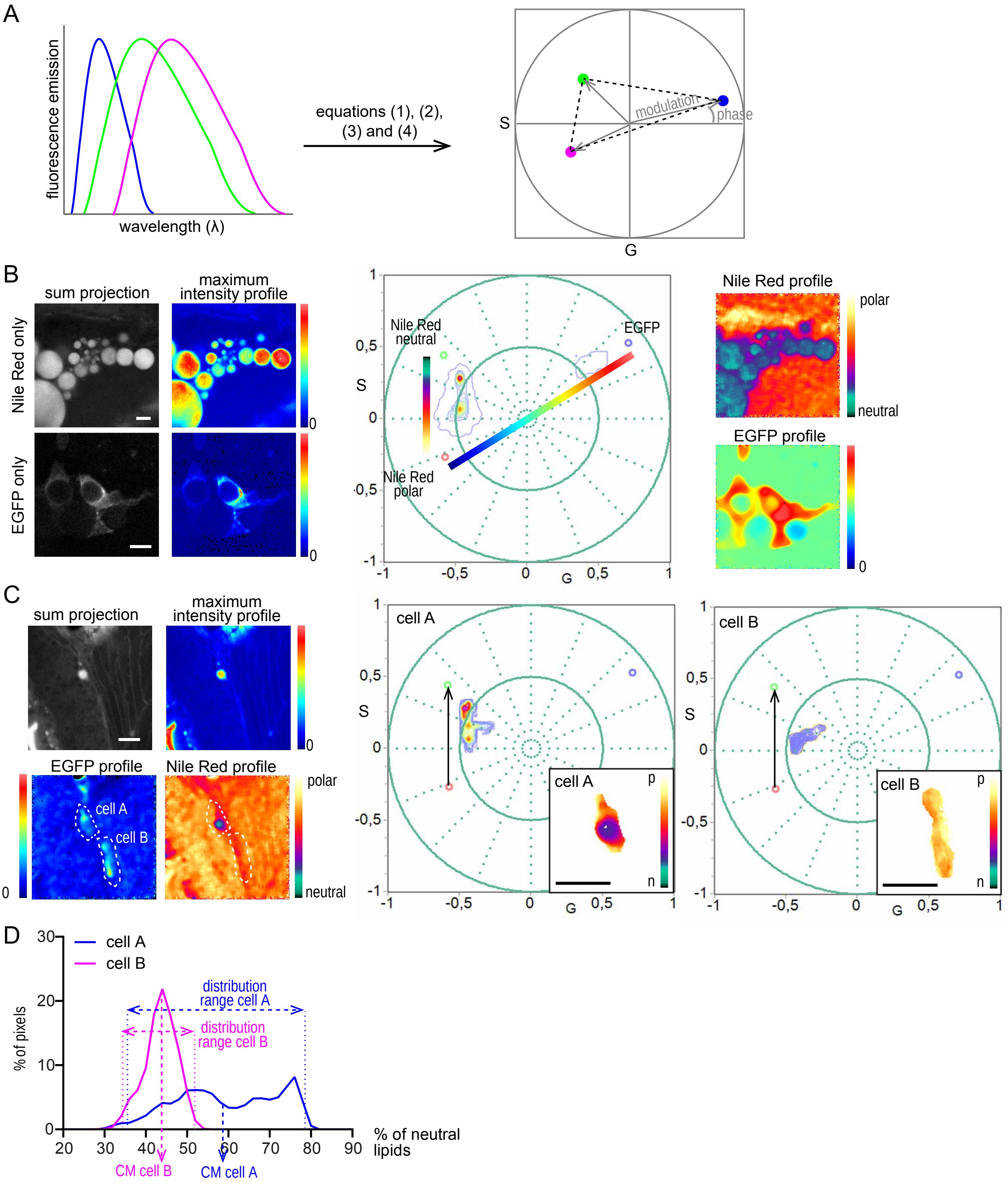

Biology Open • Supplementary information

Figure S5. Analysis of the lipid metabolic profile of EGFP+ cells.

A. Schematic representation of the transformation of the individual spectra of three different fluorophores into the same phasor plot using equations (1) to (4) indicated in "Materials and Methods" section. Dashed lines indicate the area (triangle) in the phasor plot in which pixels with the combination of the three different fluorophores will appear.

B. Examples of hyperspectral images of control cells and the localization of each pixel in a phasor plot. Images in the left are projections of spectral images and were colored according to pixel intensity maximum. Images of wild type larvae stained with Nile Red ("Nile Red only") lay within a line-shaped trajectory, corresponding to regions of different lipid polarity within the cell, were used to define the position of two of the components (Nile Red in a polar environment: red circle; Nile Red in a neutral environment: green circle). Instead, images of cells in *fabp4a(-2.7):EGFPcaax* larvae without staining ("EGFP only") appear in a defined region with low phase angle, which was used to define the position of the third component (EGFP: blue circle). The images in the right were colored according to the position of pixels in the phasor plot (the color scales were superimposed to the Nile Red trajectory and the EGFP axis for improving clarity).

C. Example of cells in a *fabp4a(-2.7):EGFPcaax* larvae of 8 dpf stained with Nile Red. Images were colored according to pixel intensity or to the position of pixels in the phasor plots. Phasor plots corresponding to the thresholded cells are presented in the right side. The direction of the Nile Red axis used for plots in (D) is denoted by a black arrow.

D. Normalized distribution of pixels along the Nile Red axis in the phasor plot for the cells in (C). The "Nile Red axis" corresponds to the percentage of polar lipids in the region of the cell analyzed. The center of mass and range of these distributions were calculated as described in the "Material and methods" section and are schematically represented in the plot. Scale bars: B: 20 µm; C: 50 µm.

Biology Open • Supplementary information

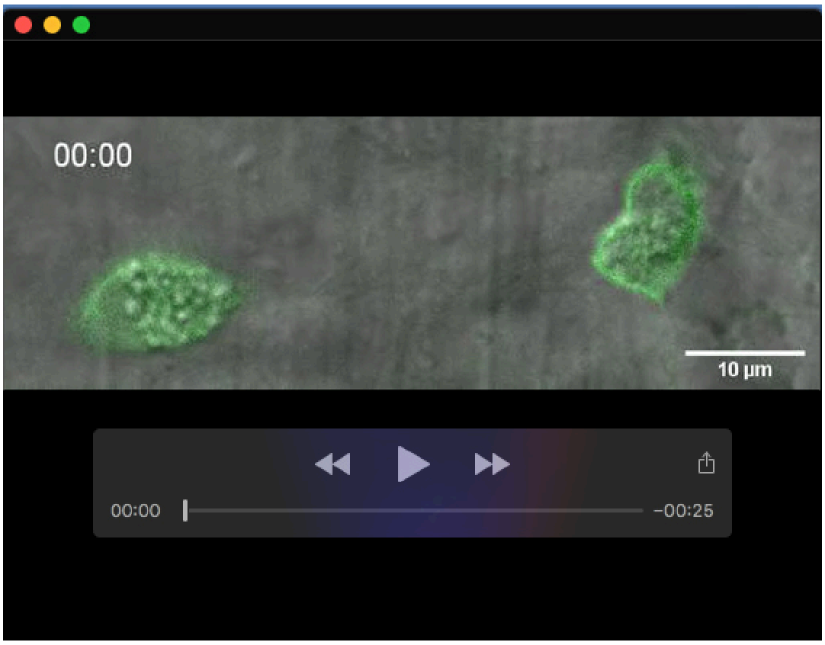

Movie 1. Time-lapse imaging of EGFP+/LD- cells. The EGFPcaax signal is in green and the transmitted light in grey. Time is showed in minutes:seconds format. Scale bar: 10 µm.

Biology Open • Supplementary information
